# Combined drug therapy with ultrasound-guided stellate ganglion block for primary tinnitus

**DOI:** 10.1097/MD.0000000000048044

**Published:** 2026-03-20

**Authors:** Zhifu Chen, Juan Zhang, Tao Bai, Peirong Yang, Guochang Song, Anjun Yan, Ruiling Wang

**Affiliations:** a Department of Anesthesiology, Bao Ji People’s Hospital, Baoji City, Shaanxi Province, China; b Department of Ultrasonic Medicine, Baoji Third People’s Hospital, Baoji City, Shaanxi Province, China; c Department of Endemic Disease Control, Baoji City Center for Disease Control and Prevention, Baoji City, Shaanxi Province, China; d Department of Otolaryngology, Bao Ji People’s Hospital, Baoji City, Shaanxi Province, China.

**Keywords:** drug therapy, primary tinnitus, quality of life, severity, ultrasound-guided stellate ganglion block

## Abstract

This study aimed to evaluate the effectiveness and safety of combined medication therapy and ultrasound-guided stellate ganglion block treatment for primary tinnitus. This retrospective cohort study reviewed the clinical data of 113 patients with primary tinnitus, with 46 patients receiving stellate ganglion block treatment as the control group and 67 patients receiving the combined medication therapy combined with ultrasound-guided stellate ganglion block treatment as the observation group. Baseline characteristics, tinnitus severity scores, quality of life, psychological health, adverse events, and patient satisfaction were assessed and compared between the 2 treatment groups. Baseline demographics and tinnitus characteristics were similar between groups (*P* > .05). Posttreatment, the observation group showed significantly greater improvements in tinnitus severity (all metrics, *P* < .05), quality of life (physical, emotional, role, cognitive, social domains, *P* < .005), and psychological health (anxiety, depression, stress, sleep, social support, *P* < .05). Adverse events (headache, dizziness, nausea) were comparable (*P* > .05). Patient satisfaction favored the observation group (*P* = .034), though fewer reported “very satisfied”. The combined intervention demonstrates superior efficacy across clinical and psychosocial outcomes with comparable safety. Medication therapy combined with ultrasound-guided stellate ganglion block treatment significantly reduces tinnitus severity. However, due to the retrospective observational nature of this study, the findings demonstrate an association rather than a causal relationship.

## 1. Introduction

Tinnitus, the perception of sound in the absence of external auditory stimuli, is a common and distressing symptom frequently encountered in clinical practice.^[1,2]^ The prevalence of tinnitus in adults is approximately 10%–15%, with about 1%–3% experiencing a severe impact on their quality of life due to intense tinnitus.^[3–5]^ Despite its high prevalence and impact on individual health, there is currently no universally effective treatment for tinnitus.

Current treatment options include sound therapy, cognitive behavioral therapy, and medication; however, these treatments typically offer limited efficacy.^[6–8]^ The stellate ganglion comprises the sixth and seventh cervical vertebrae as well as the first thoracic sympathetic ganglion, functionally belonging to the sympathetic nerve. Stellate ganglion block (SGB), a well-established anesthesia technique, is performed for the treatment of head, face, neck, and upper limb pain.^[9]^ Some studies suggest that the potential role of overactive sympathetic nervous system in the pathogenesis of tinnitus has increased interest in sympathetic nerve blockade as a therapeutic approach. The overexcitation of the sympathetic nervous system not only leads to increased sensitivity in the auditory central nervous system but also causes constriction of vascular components around the ear, resulting in a decrease in blood flow and velocity, thus triggering or exacerbating tinnitus. This mechanism is supported by recent studies linking sympathetic overactivity to cochlear hypoperfusion and tinnitus generation.^[10–12]^ Therefore, employing a SGB for tinnitus treatment aims to reduce the vasoconstriction in the innervated region by this nerve, expand cerebral blood vessels, increase cochlear blood volume, and ultimately ameliorate tinnitus. SGB’s potential efficacy in the treatment of tinnitus has recently been explored.^[13–15]^

Medication therapy (MT) is a common treatment approach currently employed, yet there is no standardized pharmacological model for managing tinnitus.^[16]^ Clinicians treating chronic tinnitus may utilize a variety of medications such as antidepressants, benzodiazepines, non-benzodiazepine anticonvulsants, glutamate receptor antagonists, dopamine agonists and antagonists, or muscle relaxants. However, tinnitus is largely a subjective symptom with diverse etiologies and clinical presentations, including varying degrees of psychiatric symptoms. Consequently, there is currently no definitive pharmacological model in clinical practice for treating tinnitus comprehensively. But, the use of combined medication therapy combined with ultrasound-guided stellate ganglion block (MTSGB) shows promise in addressing the neurophysiological and psychological aspects of tinnitus, with potential impact on the condition.^[17,18]^

In this context, we conducted a retrospective cohort study to evaluate the effectiveness and safety of combined MTSGB treatment for primary tinnitus. The main objective of this study was to assess changes in the severity of tinnitus, quality of life, and psychological well-being following the intervention.

## 2. Materials and methods

### 2.1. Study design and participants

This study was approved by the Ethics Committee of Bao Ji People’s Hospital. This retrospective analysis reviewed the clinical data of 113 patients with primary tinnitus who visited the outpatient departments of otolaryngology or pain management at our hospital from March 2021 to December 2023. The patients were divided based on treatment method into the ultrasound-guided stellate ganglion block (USGB) treatment group (the control group, n = 46) and the combined MTSGB group (the observation group, n = 67).

This study was approved by Ethics Committee of the Hospital. All procedures performed in studies involving human participants were in accordance with the ethical standards of the institutional and/or national research committee and with the 1964 Helsinki declaration and its later amendments or comparable ethical standards. Informed consent was waived for this retrospective study due to the exclusive use of de-identified patient data, which posed no potential harm or impact on patient care.

### 2.2. Inclusion criteria and exclusion criteria

Inclusion criteria were as followed: patients diagnosed with primary tinnitus by associate chief physicians of otolaryngology or pain management and above, based on relevant diagnostic criteria^[19]^; patients who have a correct understanding of and complete the questionnaire, and can complete the relevant forms under the guidance of medical staff.

Exclusion criteria were as followed: patients with organic lesions indicated by sound adaptation test, vestibular function test, or cranial magnetic resonance imaging, and those diagnosed with secondary tinnitus based on auxiliary examinations, such as acoustic neuroma^[20,21]^; patients found to have tinnitus caused by otitis media, tympanic membrane perforation, or other conditions following specialized examinations in otolaryngology and other disciplines^[22,23]^; patients with severe cognitive dysfunction who are unable to cooperate with treatment^[24–26]^; patients with coagulation abnormalities or severe cardiovascular and cerebrovascular diseases^[27,28]^; patients with severe hypertension, diabetes, and inadequate recent blood pressure or glucose control; patients who are unable to adhere to the treatment schedule during the therapeutic period; pregnant or breastfeeding women; patients allergic to the medications used; patients who have received other types of treatment.

### 2.3. Intervention

The treatment approach for the control group involves the utilization of USGB therapy. Lidocaine injection, concentration 1%, dose 5 ml, once daily, for a course of 7 days.

The treatment approach for the observation group involves the utilization of combined MT with USGB therapy. Concurrently with USGB therapy, pharmacological treatment was administered. This included oral administration of 0.5 mg methylcobalam in tablets 3 times daily, *Ginkgo biloba* tablets at 0.19 g per tablet, taken orally at a dosage of 2 tablets 3 times daily, and Wuling capsules at 0.33 g per capsule, taken orally at a dosage of 3 capsules 3 times daily. The medication regimen was adhered to continuously for 2 weeks.

SGB was administered ipsilateral to tinnitus laterality. Successful blockade was confirmed by Horner syndrome (ptosis/miosis). Audiometric evaluation (pure-tone thresholds) was performed at baseline but not repeated posttreatment, as outcomes relied on patient-reported scales.

The required instruments for the study included: portable ultrasound machine (Sonosite M-Turbo, linear array transducer 5–12 MHz); single-use sterile injection needles (Zhejiang Kindly Medical Devices Co., Ltd., Wenzhou, Zhejiang, China; registration No. 20193141818); single-use sterile syringes (Jiangsu Kangjin Medical Instrument Co., Ltd., Changzhou, Jiangsu, China; registration No. 20143142098). The necessary medications for the study were: 0.9% sodium chloride injection (Shijiazhuang No. 4 Pharmaceutical Co., Ltd., Shijiazhuang, Hebei, China; approval No. H13023202); 2% lidocaine hydrochloride injection (Shanghai Hefeng Pharmaceutical Co., Ltd., Shanghai, China; approval No. H20023777); mecobalamin tablets, 0.5 mg per tablet (Eisai China Pharmaceutical Co., Ltd., Suzhou, Jiangsu, China; approval No. H20143107); *G biloba* tablets, 0.19 g per tablet (Sichuan Senke Pharmaceutical Co., Ltd., Meishan, Sichuan, China; approval No. Z20063496); Wuling capsules, 0.33 g per capsule (Zhejiang Jolly Pharmaceutical Co., Ltd., Deqing, Zhejiang, China; approval No. Z19990048). The required safety equipment for the study included: airway management supplies (oxygen suction tubing, laryngoscope, tracheal tube, oropharyngeal or nasopharyngeal airway, laryngeal mask, dental pad, and pressure breathing bag); sputum suction device, suction catheter, and negative pressure suction equipment; resuscitation vehicle, including emergency supplies and medications; monitor, oxygen source, and ventilator.

### 2.4. Observed parameters

Baseline characteristics, Tinnitus Severity Scores, Quality of Life, Psychological Health Assessment, Adverse Events. Assessments were performed at 2 weeks after treatment initiation. A satisfaction survey was conducted to obtain overall patient evaluations of the treatment, categorized as very satisfied, satisfied, or dissatisfied.

#### 2.4.1. Tinnitus severity scores

Tinnitus handicap inventory assessment questionnaire (0–100) spans 3 main domains: functional, emotional, and cognitive. The reliability of this questionnaire is reported to be between 0.78 and 0.90.^[29]^ Tinnitus functional index (0–100) is a comprehensive tool incorporating various modules designed to evaluate the severity of tinnitus and its associated negative impacts. The reliability of this scale is reported at 0.97.^[30]^ Tinnitus visual analog scale, (VAS, 0–10) was utilized to assess the patients’ pain. The reliability of the VAS score was 0.94.^[31]^ Tinnitus reaction questionnaire (0–104) is utilized for evaluating the loudness and distress caused by tinnitus. The reliability of this scale is reported as 0.96.^[32]^

#### 2.4.2. Quality of life

The European Organisation for Research and Treatment of Cancer Quality of Life Core Questionnaire (0–100) was used to evaluate the quality of life. The Cronbach alpha coefficients for the questionnaire is 0.927, indicating acceptable internal consistency.^[33]^

#### 2.4.3. Psychological health assessment

Self-rating anxiety scale (0–100) was used to assess individuals’ anxiety levels. The reliability of the scale ranges from 0.823 to 0.868 according to the citation provided.^[34]^ Patient health questionnaire-9 (0–27) was used to assess the severity of depression. The reliability of this scale is reported to be 0.82.^[35]^ The perceived stress scale-10 (1–50) is utilized for assessing an individual’s subjective perception of stress levels and coping abilities. The reliability of this scale has been reported as 0.85.^[36]^ The athens insomnia scale (1–24) is employed to quantify sleep difficulties. The reliability coefficient of this scale is reported to be 0.90.^[37]^ The perceived social support scale (0–84) is a measure that emphasizes an individual’s self-understanding and self-perception of social support. The reliability of this scale is reported to be 0.92.^[38]^

### 2.5. Statistical analysis

The relevant data was processed using SPSS 25.0 (IBM Corp., Armonk) statistical software. Continuous data with normal distribution are presented as mean ± standard deviation and were analyzed using Student *t* test. Non-normally distributed continuous data are presented as median and interquartile range and analyzed with the Wilcoxon rank sum test. Categorical variables are presented as frequencies (percentages) and compared using the Chi-square test or Fisher exact test, where appropriate. A *P* value of < .05 indicated statistical significance. A 2-tailed *P* value of < .05 was considered statistically significant.

## 3. Results

### 3.1. Baseline characteristics

No statistically significant differences were observed between the groups before treatment in terms of age, gender distribution, tinnitus duration, tinnitus loudness, tinnitus frequency, family history of tinnitus, or tinnitus location, *P* < .05 (Table 1).

**Table 1:** Baseline characteristics of patients in both groups.

Parameter	The control group (n = 46)	The observation group (n=67)	*t*/χ ^2^	*P* value
Age (yr)	52.31 ± 5.66	53.14 ± 6.28	0.734	.465
Gender (M/F)	20/26	35/32	0.524	.469
Tinnitus duration	4.76 ± 1.25	4.96 ± 1.42	0.805	.423
Tinnitus loudness (dB)	52.63 ± 5.46	53.86 ± 6.13	1.115	.268
Tinnitus frequency (Hz)	5672.34 ± 821.47	5890.75 ± 942.15	1.307	.194
Family history			0.034	.853
Yes	21 (45.65%)	33 (49.25%)		
No	25 (54.35%)	34 (50.75%)		
Tinnitus location			0.004	.949
Left	14 (30.43%)	45 (67.16%)		
Right	32 (69.57%)	22 (32.84%)		

The patients in the control group received ultrasound-guided stellate ganglion block therapy, while the patients in the observation group received combined medication therapy combined with the block therapy. F = female, Hz = hertz, M = male, dB = decibel, n = number of participants, *t*/x2 = Student t test/Chi-square test, yr = years.

### 3.2. Tinnitus severity scores

In terms of the severity score of tinnitus, no statistically significant difference was observed between the 2 groups before treatment (*P* > .05 for all). Posttreatment, the observation group showed significantly greater improvement than the control group in all metrics: tinnitus handicap inventory (*P* = .021), tinnitus functional index (*P* = .002), VAS (*P* = .010), and tinnitus reaction questionnaire (*P* < .001) (Table 2).

**Table 2:** Comparison of tinnitus severity scores before and after treatment between 2 groups.

Parameter		The control group (n = 46)	The observation group (n = 67)	*t*	*P* value
THI (0–100)	Before	55.36 ± 8.27	55.28 ± 8.14	0.049	.961
	After	46.32 ± 7.43	43.14 ± 6.93	2.327	.021
TFI (0–100)	Before	37.24 ± 4.59	38.16 ± 4.58	1.050	.296
	After	28.25 ± 4.62	25.59 ± 4.24	3.159	.002
VAS (0–10)	Before	7.51 ± 1.33	7.60 ± 1.50	0.333	.740
	After	6.45 ± 1.23	5.85 ± 1.16	2.636	.010
TRQ (0–104)	Before	75.34 ± 9.25	76.19 ± 8.77	0.496	.621
	After	57.14 ± 6.57	50.59 ± 6.88	5.063	< .001

The patients in the control group received ultrasound-guided stellate ganglion block therapy, while the patients in the observation group received combined medication therapy combined with the block therapy. TFI = tinnitus functional index, THI = tinnitus handicap inventory, TRQ = Tinnitus Reaction Questionnaire, VAS = Visual Analog Scale., n = number of participants, *t* = Student *t* test.

### 3.3. Quality of life

The retrospective cohort study compared the effects of combined drug therapy and USGB on the quality of life of patients with primary tinnitus. There was no significant difference in quality of life between the 2 groups before treatment. Posttreatment, the observation group demonstrated significantly greater improvements than the control group in physical symptoms (*P* < .001), emotional management (*P* = .015), role playing (*P* = .021), cognitive function (*P* = .004), and social function (*P* < .001), as showed in Table 3.

**Table 3:** Comparison of quality of life before and after treatment between 2 groups.

Parameter		The control group (n = 46)	The observation group (n = 67)	*t*	*P* value
Physical symptoms	Before	63.28 ± 5.77	64.15 ± 5.68	0.794	.429
	After	75.27 ± 5.72	81.71 ± 3.27	7.584	<.001
Emotional management	Before	68.03 ± 5.24	68.34 ± 5.19	0.309	.758
	After	79.28 ± 5.18	82.01 ± 6.11	2.476	.015
Role playing	Before	63.22 ± 4.65	63.96 ± 5.68	0.727	.469
	After	79.14 ± 4.57	81.29 ± 4.97	2.341	.021
Cognitive function	Before	64.92 ± 4.37	64.88 ± 3.59	0.058	.954
	After	79.25 ± 6.32	82.31 ± 4.79	2.922	.004
Social function	Before	60.13 ± 5.68	60.55 ± 4.76	0.429	.669
	After	75.08 ± 4.07	81.21 ± 3.88	8.084	<.001

n = number of participants, *t* = Student *t* test.

### 3.4. Psychological health assessment

Compared to the control group, there was no difference between the 2 groups before treatment. After treatment, the observation group showed significant improvements versus controls: anxiety (*P* = .044), depression (*P* = .034), stress (*P* < .001), sleep quality (*P* < .001), and social support (*P* = .009), as showed in Table 4.

**Table 4:** Comparison of psychological health assessment before and after treatment between 2 groups.

Parameter		The control group (n = 46)	The observation group (n = 67)	*t*	*P* value
Anxiety score (0–100)	Before	67.66 ± 5.39	68.51 ± 5.37	0.821	.413
	After	61.53 ± 4.25	59.81 ± 4.52	2.036	.044
Depression score (0–27)	Before	20.06 ± 2.65	20.67 ± 3.11	1.076	.284
	After	14.75 ± 4.16	13.12 ± 3.83	2.146	.034
Stress levels (1–50)	Before	40.57 ± 2.68	40.35 ± 2.64	0.448	.655
	After	30.32 ± 1.53	25.32 ± 1.35	7.626	<.001
Sleep quality (1–24)	Before	8.24 ± 1.65	8.76 ± 1.21	1.797	.076
	After	9.13 ± 1.74	12.62 ± 1.86	6.534	<.001
Social support score (0–84)	Before	50.01 ± 5.34	51.20 ± 5.64	1.130	.261
	After	60.23 ± 8.54	65.35 ± 7.66	2.680	.009

n = number of participants, *t* = Student *t* test.

### 3.5. Adverse events

There were no statistically significant differences between the 2 groups in the incidence of headache (6.52% in USGB vs 7.46% in Drug Therapy with Ultrasound-Guided Stellate Ganglion Block [DTUSGB], *P* = 1), dizziness (13.04% in USGB vs 14.93% in DTUSGB, *P* = .994), and nausea (2.17% in USGB vs 8.96% in DTUSGB, *P* = .284), as shown in Table 5.

**Table 5:** Comparison of adverse events between 2 groups.

Parameter	The control group (n = 46)	The observation group (n = 67)	χ ^2^	*P* value
Headache	3 (6.52%)	5 (7.46%)	0	1
Dizziness	6 (13.04%)	10 (14.93%)	0	.994
Nausea	1 (2.17%)	6 (8.96%)	1.149	.284

n = number of participants, χ 2 = Chi-square test.

### 3.6. Patient satisfaction

A statistically significant result indicated a higher level of satisfaction in the observation group compared to the control group (χ 2 = 6.734, *P* = .034). The observation group had fewer “Very Satisfied” (43.48% vs 56.72%) but more “Somewhat Satisfied” (52.17% vs 29.85%) and fewer “Not Satisfied” (4.35% vs 13.43%) compared to controls, as shown in Table 6.

**Table 6:** Comparison of patient satisfaction between 2 groups.

Satisfaction level	The control group (n = 46)	The observation group (n = 67)	χ 2	*P* value
Very satisfied	26 (56.72%)	29 (43.48%)	6.734	.034
Somewhat satisfied	14 (29.85%)	35 (52.17%)		
Not satisfied	6 (13.43%)	3 (4.35%)		

n = number of participants, χ 2 = Chi-square test.

## 4. Discussion

The medications utilized in this study comprise methylcobalamin, *G biloba* leaf extract, and Wuling capsules. Methylcobalamin, acting as an endogenous coenzyme, enters neuronal cell organelles in the human brain to participate in the synthesis of neuronal thymidine nucleosides, accelerating nerve repair and thereby ameliorating tinnitus symptoms. *G biloba* leaf extract, a traditional Chinese medicine, primarily functions to invigorate the blood, dispel stasis, and promote circulation. Its key component, *G biloba* leaf extract, possesses pharmacological effects of dilating coronary arteries, counteracting adrenaline-induced vasoconstriction, increasing cerebral blood flow, reducing vascular resistance, exhibiting potent antiplatelet aggregation effects, and effectively harmonizing renal qi post-administration; gradually alleviating tinnitus symptoms in accordance with traditional Chinese medical theories. Wuling capsules contain Auricularia auricula-judae mycelium as the main ingredient, a rare medicinal fungus in China encompassing various amino acids such as glutamic acid and γ-aminobutyric acid, vitamins, trace elements, and other nutritional components.^[39–41]^ When combined with dexamethasone, it regulates endocrine functions, enhances immune capabilities, provides efficient sedative effects on the central nervous system, and significantly aids in treating anxiety and depressive moods. The combined drug therapy, accompanied by SGB treatment, synergistically enhances the alleviation of tinnitus symptoms in patients, as evidenced by the findings in this study. SGB dilates cerebral vasculature and increases cochlear blood flow,^[42]^ while *G biloba* potentiates vasodilation via platelet-activating factor antagonism.^[43]^ Methylcobalamin accelerates nerve repair,^[44]^ synergizing with SGB’s modulation of sympathetic overactivity. In this study, the observed reductions in tinnitus severity scores are indicative of the effectiveness of combined MT alongside SGB treatment in alleviating the subjective experience of tinnitus, which is crucial for improving the overall well-being of individuals affected by this condition. The study of Holgers KM et al also supports this view.^[45]^

The drug therapy combined group demonstrated significantly higher scores in quality of life and psychological health of patients with primary tinnitus suggest that the combined drug therapy may offer advantages over SGB alone in improving the quality of life in patients with primary tinnitus. Additionally, the study revealed significant differences in various psychological indicators, with the drug therapy combined group demonstrating favorable outcomes in terms of lower anxiety scores, lower depression scores, lower stress levels, improved sleep quality, enhanced cognitive functioning, and higher social support scores. When patients suffer from tinnitus, especially when it is persistent, many individuals find themselves in distress and confusion. This may lead to a range of psychological issues including anxiety, worry, irritability, depression, and even fear. These psychological challenges can further exacerbate tinnitus, creating a vicious cycle. As tinnitus symptoms alleviate, the patient’s psychological concerns also tend to alleviate accordingly. The study by Kang DW et al also revealed the extent to which psychological indicators can improve tinnitus.^[46]^

When considering the safety profile of the 2 treatment modalities, the incidence of adverse events was slightly higher in the DTUSGB group compared to the USGB group. However, there were no statistically significant differences in the occurrence of adverse events between the groups, indicating a comparable safety profile for the 2 treatment approaches. Furthermore, the study demonstrated significant differences in patient satisfaction between the 2 groups, with the USGB group showing notably higher rates of “very satisfied” responses compared to the drug therapy combined group. These findings highlight the preference for USGB among patients with primary tinnitus, indicating a higher level of satisfaction with this treatment modality. Higher satisfaction in the SGB-only group may reflect procedural simplicity despite superior efficacy of combined therapy. Patient preferences for minimally invasive treatments warrant consideration.^[47]^

However, several limitations of the study should be considered. As a retrospective study, our findings indicate associations rather than causation. Future prospective trials are needed to establish causality. The retrospective nature of the analysis may have introduced bias, and the relatively small sample size warrants cautious interpretation of the results. Future prospective studies with larger sample sizes and longer follow-up durations are needed to further elucidate the comparative effectiveness and long-term outcomes of the 2 treatment modalities. Besides, assessments captured short-term outcomes (2 weeks). Long-term efficacy requires prospective trials with extended follow-up. Additionally, the absence of a control group receiving only medications without the USGB could limit the interpretation of the specific contribution to the observed outcomes. The lack of a medication-only group limits isolation of SGB’s specific contribution. Future studies should include this arm. Exploration of potential mechanisms underlying the observed treatment effects, such as modulation of sympathetic nervous system activity, and neurophysiological and psychological aspects of tinnitus, is warranted to enhance our understanding of the therapeutic potential of combined MT and USGB for primary tinnitus. It should be emphasized that, as a retrospective study, residual confounding cannot be fully excluded despite baseline comparisons between groups. Second, multivariable adjustment for all potential confounders was limited by the available clinical data, and unmeasured factors such as psychological stress, sleep quality, or concurrent therapies may have influenced the outcomes. Third, the absence of longitudinal modeling restricts the ability to evaluate dynamic changes over time. These limitations may affect the robustness of the observed associations.

## 5. Conclusion

Combined MT and USGB treatment for primary tinnitus can significantly reduce tinnitus severity, improve quality of life, and enhance psychological well-being. The results show that the medications played a crucial role in alleviating tinnitus symptoms and improving psychological indicators. Compared to the USGB treatment alone, the combined therapy group exhibited significantly lower tinnitus severity scores, reduced anxiety and depression levels, decreased stress, improved sleep quality, and enhanced cognitive function. Despite this, patient satisfaction was higher in the group receiving only the USGB, suggesting that individual patient preferences should be considered in clinical practice. These results suggest a potential association between DTUSGB and improved tinnitus outcomes; however, causal inferences cannot be drawn without prospective randomized controlled trials.

## Acknowledgements

The authors express their appreciation to staff in Bao Ji People’s Hospital, Baoji Third People’s Hospital and Baoji City Center for Disease Control and Prevention, for their technical assistance.

## Author contributions

**Conceptualization:** Zhifu Chen, Juan Zhang, Tao Bai, Peirong Yang, Guochang Song, Anjun Yan, Ruiling Wang.

**Data curation:** Zhifu Chen, Juan Zhang, Tao Bai, Peirong Yang, Guochang Song, Anjun Yan, Ruiling Wang.

**Formal analysis:** Zhifu Chen, Juan Zhang, Tao Bai, Peirong Yang, Guochang Song, Anjun Yan, Ruiling Wang.

**Funding acquisition:** Zhifu Chen, Juan Zhang, Peirong Yang, Anjun Yan, Ruiling Wang.

**Investigation:** Ruiling Wang.

**Writing – original draft:** Ruiling Wang.

**Writing – review & editing:** Guochang Song, Anjun Yan, Ruiling Wang.
